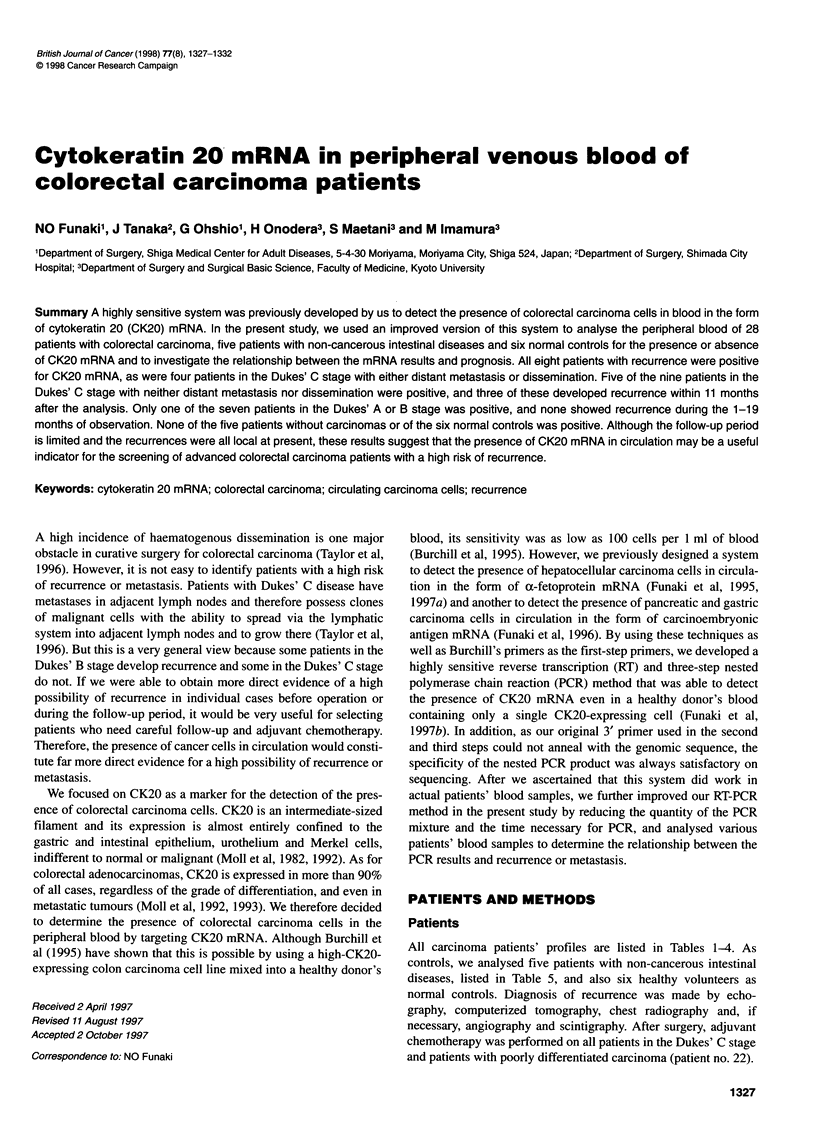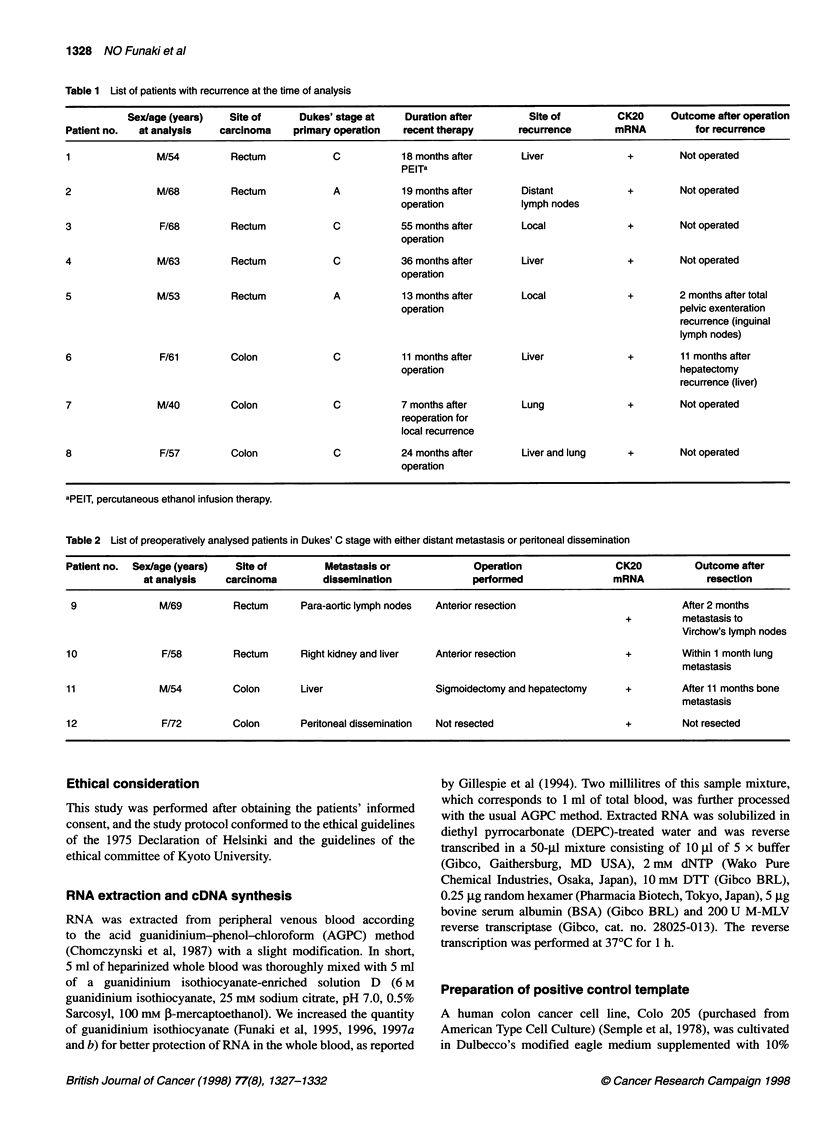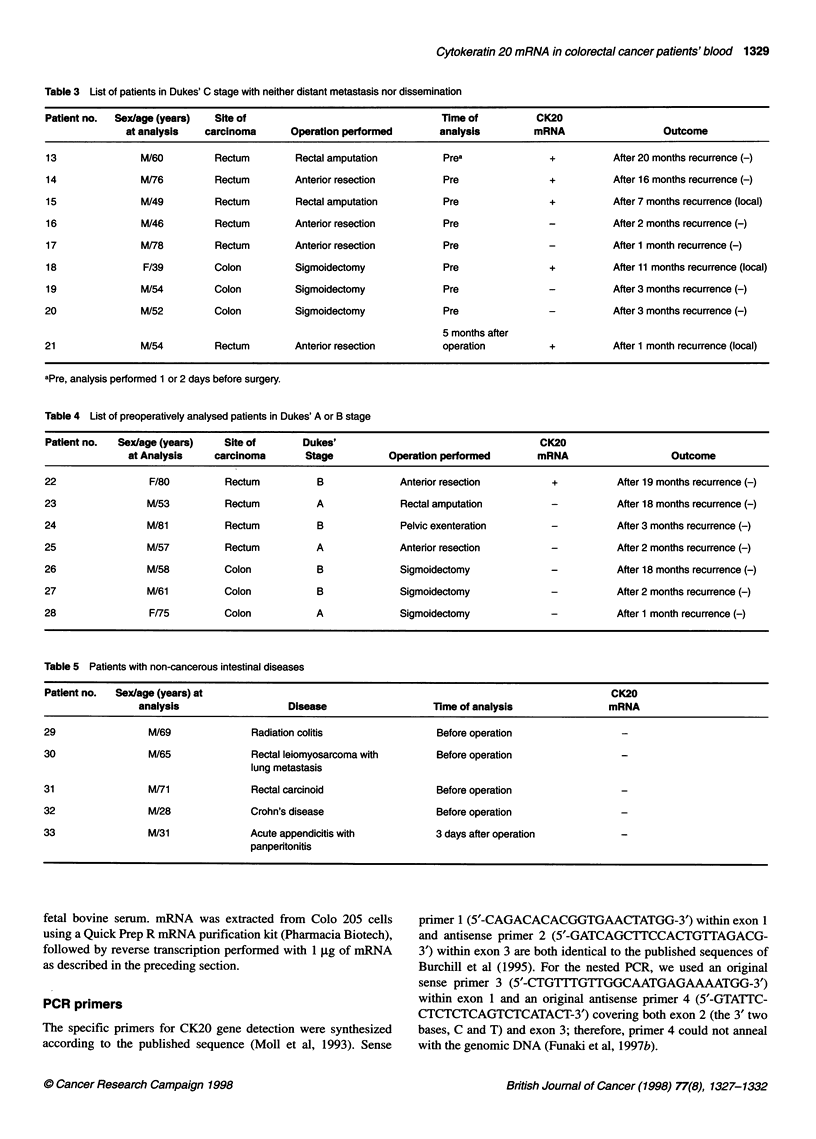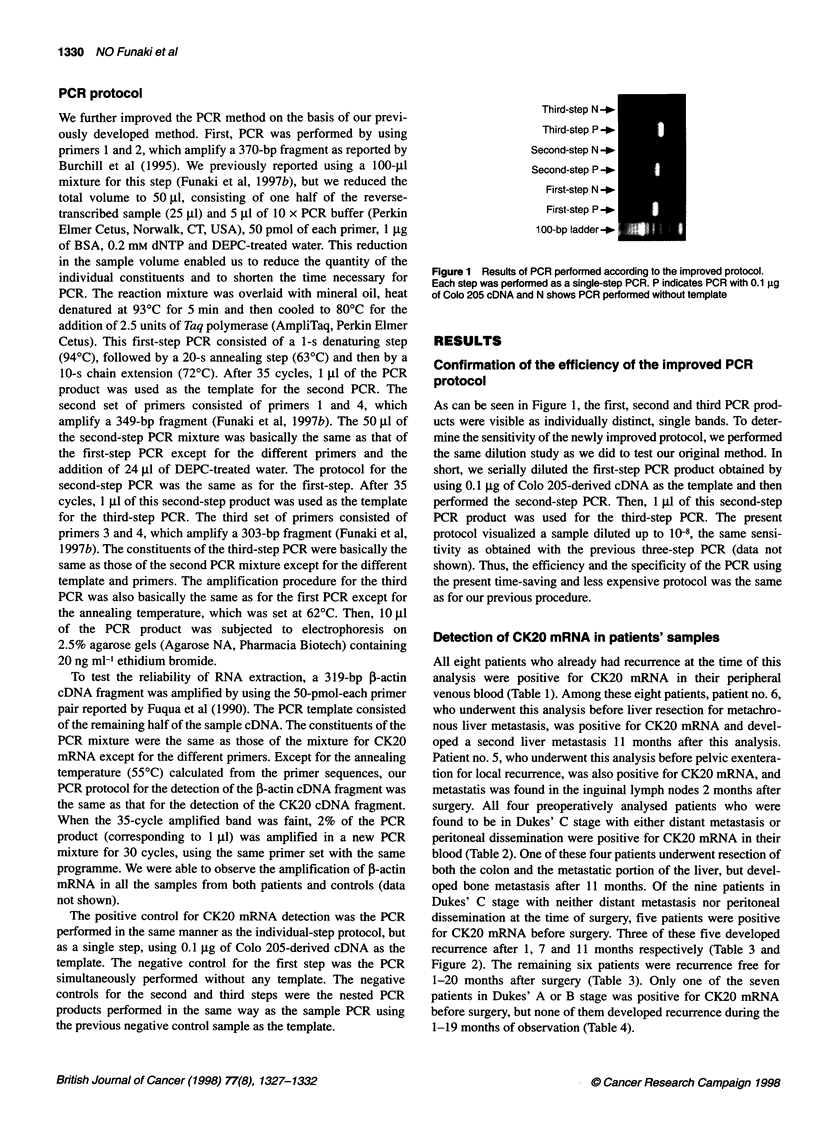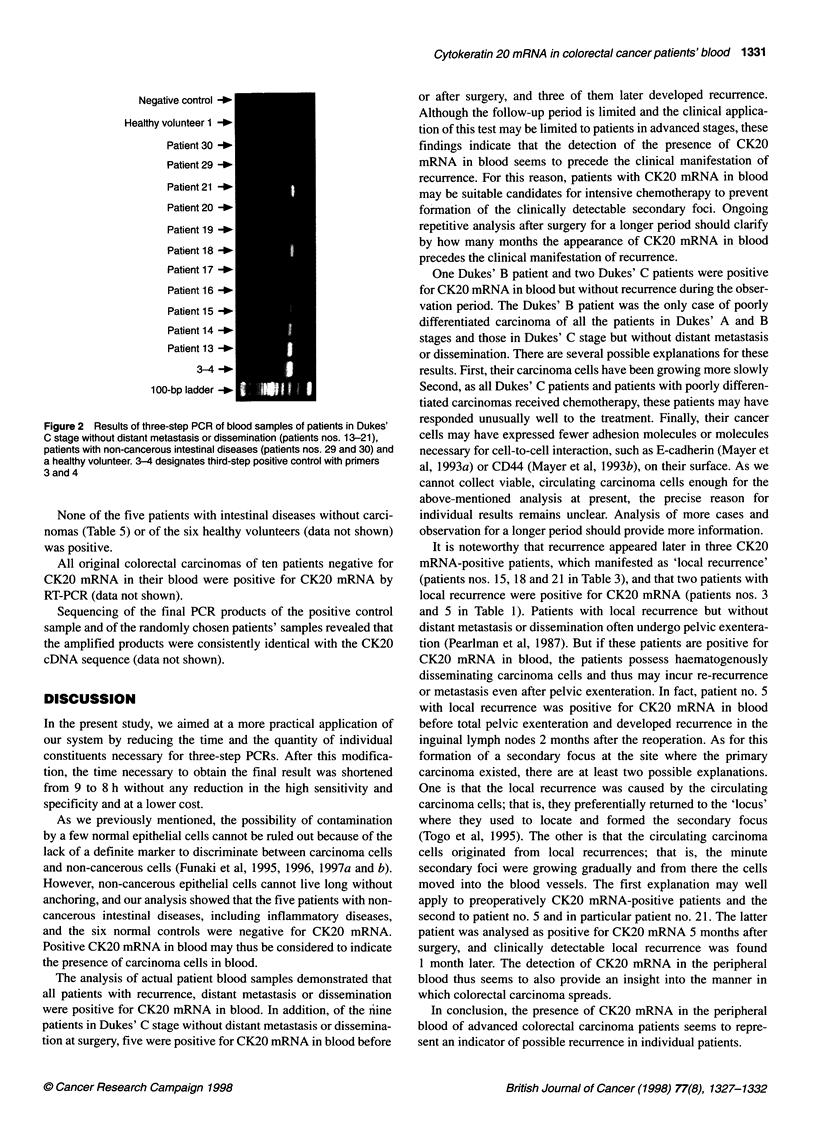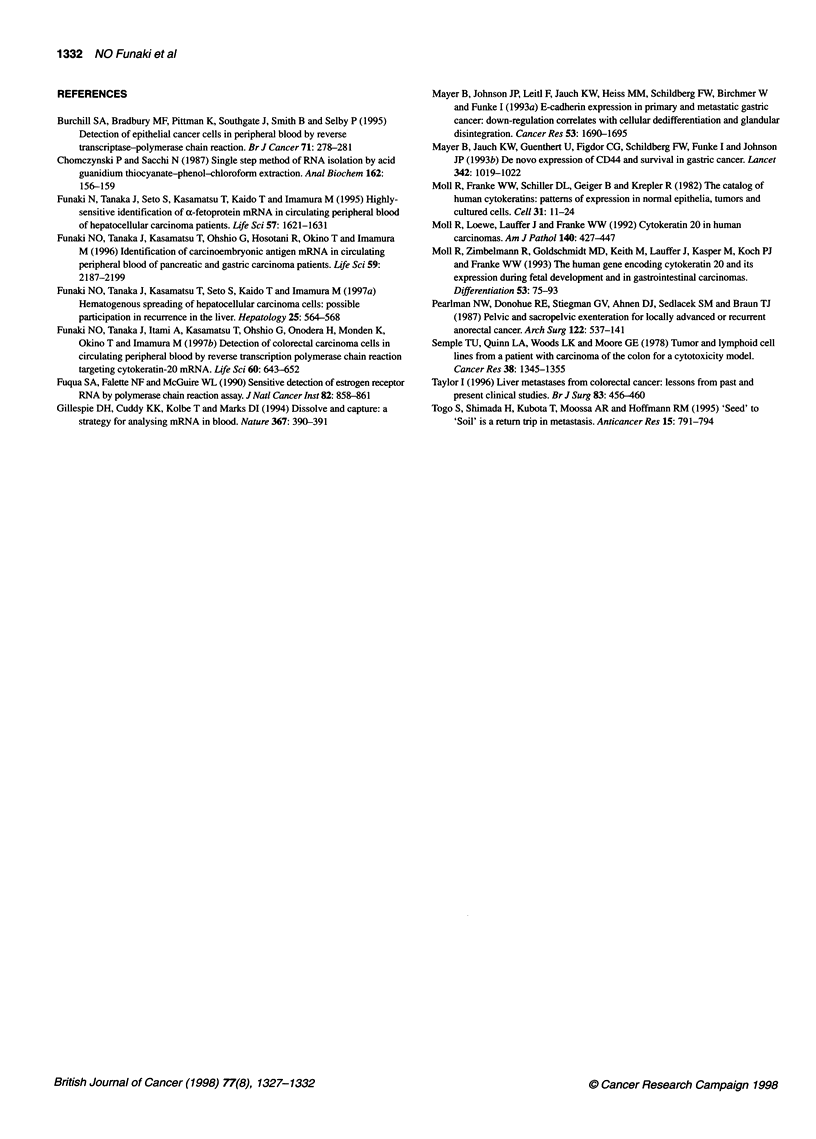# Cytokeratin 20 mRNA in peripheral venous blood of colorectal carcinoma patients.

**DOI:** 10.1038/bjc.1998.221

**Published:** 1998-04

**Authors:** N. O. Funaki, J. Tanaka, G. Ohshio, H. Onodera, S. Maetani, M. Imamura

**Affiliations:** Department of Surgery, Shiga Medical Center for Adult Diseases, Moriyama City, Japan.

## Abstract

**Images:**


British Journal of Cancer (1998) 77(8), 1327–1332
© 1998 Cancer Research Campaign

### Cytokeratin 20 mRNA in peripheral venous blood of colorectal carcinoma patients

NO Funaki[1], J Tanaka[2], G Ohshio[1], H Onodera[3], S Maetani[3] and M Imamura[3]

[1]Department of Surgery, Shiga Medical Center for Adult Diseases, 5-4-30 Moriyama, Moriyama City, Shiga 524, Japan; [2]Department of Surgery, Shimada City Hospital; [3]Department of Surgery and Surgical Basic Science, Faculty of Medicine, Kyoto University

**Summary** A highly sensitive system was previously developed by us to detect the presence of colorectal carcinoma cells in blood in the form of cytokeratin 20 (CK20) mRNA. In the present study, we used an improved version of this system to analyse the peripheral blood of 28 patients with colorectal carcinoma, five patients with non-cancerous intestinal diseases and six normal controls for the presence or absence of CK20 mRNA and to investigate the relationship between the mRNA results and prognosis. All eight patients with recurrence were positive for CK20 mRNA, as were four patients in the Dukes' C stage with either distant metastasis or dissemination. Five of the nine patients in the Dukes' C stage with neither distant metastasis nor dissemination were positive, and three of these developed recurrence within 11 months after the analysis. Only one of the seven patients in the Dukes' A or B stage was positive, and none showed recurrence during the 1–19 months of observation. None of the five patients without carcinomas or of the six normal controls was positive. Although the follow-up period is limited and the recurrences were all local at present, these results suggest that the presence of CK20 mRNA in circulation may be a useful indicator for the screening of advanced colorectal carcinoma patients with a high risk of recurrence.

**Keywords:** cytokeratin 20 mRNA; colorectal carcinoma; circulating carcinoma cells; recurrence

A high incidence of haematogenous dissemination is one major obstacle in curative surgery for colorectal carcinoma (Taylor et al, 1996). However, it is not easy to identify patients with a high risk of recurrence or metastasis. Patients with Dukes' C disease have metastases in adjacent lymph nodes and therefore possess clones of malignant cells with the ability to spread via the lymphatic system into adjacent lymph nodes and to grow there (Taylor et al, 1996). But this is a very general view because some patients in the Dukes' B stage develop recurrence and some in the Dukes' C stage do not. If we were able to obtain more direct evidence of a high possibility of recurrence in individual cases before operation or during the follow-up period, it would be very useful for selecting patients who need careful follow-up and adjuvant chemotherapy. Therefore, the presence of cancer cells in circulation would constitute far more direct evidence for a high possibility of recurrence or metastasis.

We focused on CK20 as a marker for the detection of the presence of colorectal carcinoma cells. CK20 is an intermediate-sized filament and its expression is almost entirely confined to the gastric and intestinal epithelium, urothelium and Merkel cells, indifferent to normal or malignant (Moll et al, 1982, 1992). As for colorectal adenocarcinomas, CK20 is expressed in more than 90% of all cases, regardless of the grade of differentiation, and even in metastatic tumours (Moll et al, 1992, 1993). We therefore decided to determine the presence of colorectal carcinoma cells in the peripheral blood by targeting CK20 mRNA. Although Burchill et al (1995) have shown that this is possible by using a high-CK20-expressing colon carcinoma cell line mixed into a healthy donor's blood, its sensitivity was as low as 100 cells per 1 ml of blood (Burchill et al, 1995). However, we previously designed a system to detect the presence of hepatocellular carcinoma cells in circulation in the form of α-fetoprotein mRNA (Funaki et al, 1995, 1997*a*) and another to detect the presence of pancreatic and gastric carcinoma cells in circulation in the form of carcinoembryonic antigen mRNA (Funaki et al, 1996). By using these techniques as well as Burchill's primers as the first-step primers, we developed a highly sensitive reverse transcription (RT) and three-step nested polymerase chain reaction (PCR) method that was able to detect the presence of CK20 mRNA even in a healthy donor's blood containing only a single CK20-expressing cell (Funaki et al, 1997*b*). In addition, as our original 3′ primer used in the second and third steps could not anneal with the genomic sequence, the specificity of the nested PCR product was always satisfactory on sequencing. After we ascertained that this system did work in actual patients' blood samples, we further improved our RT-PCR method in the present study by reducing the quantity of the PCR mixture and the time necessary for PCR, and analysed various patients' blood samples to determine the relationship between the PCR results and recurrence or metastasis.

*Received 2 April 1997*
*Revised 11 August 1997*
*Accepted 2 October 1997*

*Correspondence to:* NO Funaki

### PATIENTS AND METHODS

### Patients

All carcinoma patients' profiles are listed in Tables 1–4. As controls, we analysed five patients with non-cancerous intestinal diseases, listed in Table 5, and also six healthy volunteers as normal controls. Diagnosis of recurrence was made by echography, computerized tomography, chest radiography and, if necessary, angiography and scintigraphy. After surgery, adjuvant chemotherapy was performed on all patients in the Dukes' C stage and patients with poorly differentiated carcinoma (patient no. 22).

**Table 1** List of patients with recurrence at the time of analysis

| Patient no. | Sex/age (years) at analysis | Site of carcinoma | Dukes' stage at primary operation | Duration after recent therapy | Site of recurrence | CK20 mRNA | Outcome after operation for recurrence |
|---|---|---|---|---|---|---|---|
| 1 | M/54 | Rectum | C | 18 months after PEIT[a] | Liver | + | Not operated |
| 2 | M/68 | Rectum | A | 19 months after operation | Distant lymph nodes | + | Not operated |
| 3 | F/68 | Rectum | C | 55 months after operation | Local | + | Not operated |
| 4 | M/63 | Rectum | C | 36 months after operation | Liver | + | Not operated |
| 5 | M/53 | Rectum | A | 13 months after operation | Local | + | 2 months after total pelvic exenteration recurrence (inguinal lymph nodes) |
| 6 | F/61 | Colon | C | 11 months after operation | Liver | + | 11 months after hepatectomy recurrence (liver) |
| 7 | M/40 | Colon | C | 7 months after reoperation for local recurrence | Lung | + | Not operated |
| 8 | F/57 | Colon | C | 24 months after operation | Liver and lung | + | Not operated |

[a]PEIT, percutaneous ethanol infusion therapy.

**Table 2** List of preoperatively analysed patients in Dukes' C stage with either distant metastasis or peritoneal dissemination

| Patient no. | Sex/age (years) at analysis | Site of carcinoma | Metastasis or dissemination | Operation performed | CK20 mRNA | Outcome after resection |
|---|---|---|---|---|---|---|
| 9 | M/69 | Rectum | Para-aortic lymph nodes | Anterior resection | + | After 2 months metastasis to Virchow's lymph nodes |
| 10 | F/58 | Rectum | Right kidney and liver | Anterior resection | + | Within 1 month lung metastasis |
| 11 | M/54 | Colon | Liver | Sigmoidectomy and hepatectomy | + | After 11 months bone metastasis |
| 12 | F/72 | Colon | Peritoneal dissemination | Not resected | + | Not resected |

### Ethical consideration

This study was performed after obtaining the patients' informed consent, and the study protocol conformed to the ethical guidelines of the 1975 Declaration of Helsinki and the guidelines of the ethical committee of Kyoto University.

### RNA extraction and cDNA synthesis

RNA was extracted from peripheral venous blood according to the acid guanidinium–phenol–chloroform (AGPC) method (Chomczynski et al, 1987) with a slight modification. In short, 5 ml of heparinized whole blood was thoroughly mixed with 5 ml of a guanidinium isothiocyanate-enriched solution D (6 M guanidinium isothiocyanate, 25 mM sodium citrate, pH 7.0, 0.5% Sarcosyl, 100 mM β-mercaptoethanol). We increased the quantity of guanidinium isothiocyanate (Funaki et al, 1995, 1996, 1997*a* and *b*) for better protection of RNA in the whole blood, as reported by Gillespie et al (1994). Two millilitres of this sample mixture, which corresponds to 1 ml of total blood, was further processed with the usual AGPC method. Extracted RNA was solubilized in diethyl pyrrocarbonate (DEPC)-treated water and was reverse transcribed in a 50-μl mixture consisting of 10 μl of 5 × buffer (Gibco, Gaithersburg, MD USA), 2 mM dNTP (Wako Pure Chemical Industries, Osaka, Japan), 10 mM DTT (Gibco BRL), 0.25 μg random hexamer (Pharmacia Biotech, Tokyo, Japan), 5 μg bovine serum albumin (BSA) (Gibco BRL) and 200 U M-MLV reverse transcriptase (Gibco, cat. no. 28025-013). The reverse transcription was performed at 37°C for 1 h.

### Preparation of positive control template

A human colon cancer cell line, Colo 205 (purchased from American Type Cell Culture) (Semple et al, 1978), was cultivated in Dulbecco's modified eagle medium supplemented with 10%

 © Cancer Research Campaign 1998

**Table 3** List of patients in Dukes' C stage with neither distant metastasis nor dissemination

| Patient no. | Sex/age (years) at analysis | Site of carcinoma | Operation performed | Time of analysis | CK20 mRNA | Outcome |
|---|---|---|---|---|---|---|
| 13 | M/60 | Rectum | Rectal amputation | Pre[a] | + | After 20 months recurrence (–) |
| 14 | M/76 | Rectum | Anterior resection | Pre | + | After 16 months recurrence (–) |
| 15 | M/49 | Rectum | Rectal amputation | Pre | + | After 7 months recurrence (local) |
| 16 | M/46 | Rectum | Anterior resection | Pre | – | After 2 months recurrence (–) |
| 17 | M/78 | Rectum | Anterior resection | Pre | – | After 1 month recurrence (–) |
| 18 | F/39 | Colon | Sigmoidectomy | Pre | + | After 11 months recurrence (local) |
| 19 | M/54 | Colon | Sigmoidectomy | Pre | – | After 3 months recurrence (–) |
| 20 | M/52 | Colon | Sigmoidectomy | Pre | – | After 3 months recurrence (–) |
| 21 | M/54 | Rectum | Anterior resection | 5 months after operation | + | After 1 month recurrence (local) |

[a]Pre, analysis performed 1 or 2 days before surgery.

**Table 4** List of preoperatively analysed patients in Dukes' A or B stage

| Patient no. | Sex/age (years) at Analysis | Site of carcinoma | Dukes' Stage | Operation performed | CK20 mRNA | Outcome |
|---|---|---|---|---|---|---|
| 22 | F/80 | Rectum | B | Anterior resection | + | After 19 months recurrence (–) |
| 23 | M/53 | Rectum | A | Rectal amputation | – | After 18 months recurrence (–) |
| 24 | M/81 | Rectum | B | Pelvic exenteration | – | After 3 months recurrence (–) |
| 25 | M/57 | Rectum | A | Anterior resection | – | After 2 months recurrence (–) |
| 26 | M/58 | Colon | B | Sigmoidectomy | – | After 18 months recurrence (–) |
| 27 | M/61 | Colon | B | Sigmoidectomy | – | After 2 months recurrence (–) |
| 28 | F/75 | Colon | A | Sigmoidectomy | – | After 1 month recurrence (–) |

**Table 5** Patients with non-cancerous intestinal diseases

| Patient no. | Sex/age (years) at analysis | Disease | Time of analysis | CK20 mRNA |
|---|---|---|---|---|
| 29 | M/69 | Radiation colitis | Before operation | – |
| 30 | M/65 | Rectal leiomyosarcoma with lung metastasis | Before operation | – |
| 31 | M/71 | Rectal carcinoid | Before operation | – |
| 32 | M/28 | Crohn's disease | Before operation | – |
| 33 | M/31 | Acute appendicitis with panperitonitis | 3 days after operation | – |

fetal bovine serum. mRNA was extracted from Colo 205 cells using a Quick Prep R mRNA purification kit (Pharmacia Biotech), followed by reverse transcription performed with 1 μg of mRNA as described in the preceding section.

### PCR primers

The specific primers for CK20 gene detection were synthesized according to the published sequence (Moll et al, 1993). Sense primer 1 (5′-CAGACACACGGTGAACTATGG-3′) within exon 1 and antisense primer 2 (5′-GATCAGCTTCCACTGTTAGACG-3′) within exon 3 are both identical to the published sequences of Burchill et al (1995). For the nested PCR, we used an original sense primer 3 (5′-CTGTTTGTTGGCAATGAGAAAATGG-3′) within exon 1 and an original antisense primer 4 (5′-GTATTC-CTCTCTCAGTCTCATACT-3′) covering both exon 2 (the 3′ two bases, C and T) and exon 3; therefore, primer 4 could not anneal with the genomic DNA (Funaki et al, 1997*b*).

© Cancer Research Campaign 1998

### PCR protocol

We further improved the PCR method on the basis of our previously developed method. First, PCR was performed by using primers 1 and 2, which amplify a 370-bp fragment as reported by Burchill et al (1995). We previously reported using a 100-μl mixture for this step (Funaki et al, 1997*b*), but we reduced the total volume to 50 μl, consisting of one half of the reverse-transcribed sample (25 μl) and 5 μl of 10 × PCR buffer (Perkin Elmer Cetus, Norwalk, CT, USA), 50 pmol of each primer, 1 μg of BSA, 0.2 mM dNTP and DEPC-treated water. This reduction in the sample volume enabled us to reduce the quantity of the individual constituents and to shorten the time necessary for PCR. The reaction mixture was overlaid with mineral oil, heat denatured at 93°C for 5 min and then cooled to 80°C for the addition of 2.5 units of *Taq* polymerase (AmpliTaq, Perkin Elmer Cetus). This first-step PCR consisted of a 1-s denaturing step (94°C), followed by a 20-s annealing step (63°C) and then by a 10-s chain extension (72°C). After 35 cycles, 1 μl of the PCR product was used as the template for the second PCR. The second set of primers consisted of primers 1 and 4, which amplify a 349-bp fragment (Funaki et al, 1997*b*). The 50 μl of the second-step PCR mixture was basically the same as that of the first-step PCR except for the different primers and the addition of 24 μl of DEPC-treated water. The protocol for the second-step PCR was the same as for the first-step. After 35 cycles, 1 μl of this second-step product was used as the template for the third-step PCR. The third set of primers consisted of primers 3 and 4, which amplify a 303-bp fragment (Funaki et al, 1997*b*). The constituents of the third-step PCR were basically the same as those of the second PCR mixture except for the different template and primers. The amplification procedure for the third PCR was also basically the same as for the first PCR except for the annealing temperature, which was set at 62°C. Then, 10 μl of the PCR product was subjected to electrophoresis on 2.5% agarose gels (Agarose NA, Pharmacia Biotech) containing 20 ng $ml^{-1}$ ethidium bromide.

To test the reliability of RNA extraction, a 319-bp β-actin cDNA fragment was amplified by using the 50-pmol-each primer pair reported by Fuqua et al (1990). The PCR template consisted of the remaining half of the sample cDNA. The constituents of the PCR mixture were the same as those of the mixture for CK20 mRNA except for the different primers. Except for the annealing temperature (55°C) calculated from the primer sequences, our PCR protocol for the detection of the β-actin cDNA fragment was the same as that for the detection of the CK20 cDNA fragment. When the 35-cycle amplified band was faint, 2% of the PCR product (corresponding to 1 μl) was amplified in a new PCR mixture for 30 cycles, using the same primer set with the same programme. We were able to observe the amplification of β-actin mRNA in all the samples from both patients and controls (data not shown).

The positive control for CK20 mRNA detection was the PCR performed in the same manner as the individual-step protocol, but as a single step, using 0.1 μg of Colo 205-derived cDNA as the template. The negative control for the first step was the PCR simultaneously performed without any template. The negative controls for the second and third steps were the nested PCR products performed in the same way as the sample PCR using the previous negative control sample as the template.

**Figure 1** Results of PCR performed according to the improved protocol. Each step was performed as a single-step PCR. P indicates PCR with 0.1 μg of Colo 205 cDNA and N shows PCR performed without template

### RESULTS

### Confirmation of the efficiency of the improved PCR protocol

As can be seen in Figure 1, the first, second and third PCR products were visible as individually distinct, single bands. To determine the sensitivity of the newly improved protocol, we performed the same dilution study as we did to test our original method. In short, we serially diluted the first-step PCR product obtained by using 0.1 μg of Colo 205-derived cDNA as the template and then performed the second-step PCR. Then, 1 μl of this second-step PCR product was used for the third-step PCR. The present protocol visualized a sample diluted up to $10^{-8}$, the same sensitivity as obtained with the previous three-step PCR (data not shown). Thus, the efficiency and the specificity of the PCR using the present time-saving and less expensive protocol was the same as for our previous procedure.

### Detection of CK20 mRNA in patients' samples

All eight patients who already had recurrence at the time of this analysis were positive for CK20 mRNA in their peripheral venous blood (Table 1). Among these eight patients, patient no. 6, who underwent this analysis before liver resection for metachronous liver metastasis, was positive for CK20 mRNA and developed a second liver metastasis 11 months after this analysis. Patient no. 5, who underwent this analysis before pelvic exenteration for local recurrence, was also positive for CK20 mRNA, and metastatis was found in the inguinal lymph nodes 2 months after surgery. All four preoperatively analysed patients who were found to be in Dukes' C stage with either distant metastasis or peritoneal dissemination were positive for CK20 mRNA in their blood (Table 2). One of these four patients underwent resection of both the colon and the metastatic portion of the liver, but developed bone metastasis after 11 months. Of the nine patients in Dukes' C stage with neither distant metastasis nor peritoneal dissemination at the time of surgery, five patients were positive for CK20 mRNA before surgery. Three of these five developed recurrence after 1, 7 and 11 months respectively (Table 3 and Figure 2). The remaining six patients were recurrence free for 1–20 months after surgery (Table 3). Only one of the seven patients in Dukes' A or B stage was positive for CK20 mRNA before surgery, but none of them developed recurrence during the 1–19 months of observation (Table 4).

© Cancer Research Campaign 1998

**Figure 2** Results of three-step PCR of blood samples of patients in Dukes' C stage without distant metastasis or dissemination (patients nos. 13–21), patients with non-cancerous intestinal diseases (patients nos. 29 and 30) and a healthy volunteer. 3–4 designates third-step positive control with primers 3 and 4

None of the five patients with intestinal diseases without carcinomas (Table 5) or of the six healthy volunteers (data not shown) was positive.

All original colorectal carcinomas of ten patients negative for CK20 mRNA in their blood were positive for CK20 mRNA by RT-PCR (data not shown).

Sequencing of the final PCR products of the positive control sample and of the randomly chosen patients' samples revealed that the amplified products were consistently identical with the CK20 cDNA sequence (data not shown).

### DISCUSSION

In the present study, we aimed at a more practical application of our system by reducing the time and the quantity of individual constituents necessary for three-step PCRs. After this modification, the time necessary to obtain the final result was shortened from 9 to 8 h without any reduction in the high sensitivity and specificity and at a lower cost.

As we previously mentioned, the possibility of contamination by a few normal epithelial cells cannot be ruled out because of the lack of a definite marker to discriminate between carcinoma cells and non-cancerous cells (Funaki et al, 1995, 1996, 1997*a* and *b*). However, non-cancerous epithelial cells cannot live long without anchoring, and our analysis showed that the five patients with non-cancerous intestinal diseases, including inflammatory diseases, and the six normal controls were negative for CK20 mRNA. Positive CK20 mRNA in blood may thus be considered to indicate the presence of carcinoma cells in blood.

The analysis of actual patient blood samples demonstrated that all patients with recurrence, distant metastasis or dissemination were positive for CK20 mRNA in blood. In addition, of the nine patients in Dukes' C stage without distant metastasis or dissemination at surgery, five were positive for CK20 mRNA in blood before or after surgery, and three of them later developed recurrence. Although the follow-up period is limited and the clinical application of this test may be limited to patients in advanced stages, these findings indicate that the detection of the presence of CK20 mRNA in blood seems to precede the clinical manifestation of recurrence. For this reason, patients with CK20 mRNA in blood may be suitable candidates for intensive chemotherapy to prevent formation of the clinically detectable secondary foci. Ongoing repetitive analysis after surgery for a longer period should clarify by how many months the appearance of CK20 mRNA in blood precedes the clinical manifestation of recurrence.

One Dukes' B patient and two Dukes' C patients were positive for CK20 mRNA in blood but without recurrence during the observation period. The Dukes' B patient was the only case of poorly differentiated carcinoma of all the patients in Dukes' A and B stages and those in Dukes' C stage but without distant metastasis or dissemination. There are several possible explanations for these results. First, their carcinoma cells have been growing more slowly Second, as all Dukes' C patients and patients with poorly differentiated carcinomas received chemotherapy, these patients may have responded unusually well to the treatment. Finally, their cancer cells may have expressed fewer adhesion molecules or molecules necessary for cell-to-cell interaction, such as E-cadherin (Mayer et al, 1993*a*) or CD44 (Mayer et al, 1993*b*), on their surface. As we cannot collect viable, circulating carcinoma cells enough for the above-mentioned analysis at present, the precise reason for individual results remains unclear. Analysis of more cases and observation for a longer period should provide more information.

It is noteworthy that recurrence appeared later in three CK20 mRNA-positive patients, which manifested as 'local recurrence' (patients nos. 15, 18 and 21 in Table 3), and that two patients with local recurrence were positive for CK20 mRNA (patients nos. 3 and 5 in Table 1). Patients with local recurrence but without distant metastasis or dissemination often undergo pelvic exenteration (Pearlman et al, 1987). But if these patients are positive for CK20 mRNA in blood, the patients possess haematogenously disseminating carcinoma cells and thus may incur re-recurrence or metastasis even after pelvic exenteration. In fact, patient no. 5 with local recurrence was positive for CK20 mRNA in blood before total pelvic exenteration and developed recurrence in the inguinal lymph nodes 2 months after the reoperation. As for this formation of a secondary focus at the site where the primary carcinoma existed, there are at least two possible explanations. One is that the local recurrence was caused by the circulating carcinoma cells; that is, they preferentially returned to the 'locus' where they used to locate and formed the secondary focus (Togo et al, 1995). The other is that the circulating carcinoma cells originated from local recurrences; that is, the minute secondary foci were growing gradually and from there the cells moved into the blood vessels. The first explanation may well apply to preoperatively CK20 mRNA-positive patients and the second to patient no. 5 and in particular patient no. 21. The latter patient was analysed as positive for CK20 mRNA 5 months after surgery, and clinically detectable local recurrence was found 1 month later. The detection of CK20 mRNA in the peripheral blood thus seems to also provide an insight into the manner in which colorectal carcinoma spreads.

In conclusion, the presence of CK20 mRNA in the peripheral blood of advanced colorectal carcinoma patients seems to represent an indicator of possible recurrence in individual patients.

© Cancer Research Campaign 1998

### REFERENCES

Burchill SA, Bradbury MF, Pittman K, Southgate J, Smith B and Selby P (1995) Detection of epithelial cancer cells in peripheral blood by reverse transcriptase–polymerase chain reaction. *Br J Cancer* **71**: 278–281

Chomczynski P and Sacchi N (1987) Single step method of RNA isolation by acid guanidium thiocyanate–phenol–chloroform extraction. *Anal Biochem* **162**: 156–159

Funaki N, Tanaka J, Seto S, Kasamatsu T, Kaido T and Imamura M (1995) Highly-sensitive identification of α-fetoprotein mRNA in circulating peripheral blood of hepatocellular carcinoma patients. *Life Sci* **57**: 1621–1631

Funaki NO, Tanaka J, Kasamatsu T, Ohshio G, Hosotani R, Okino T and Imamura M (1996) Identification of carcinoembryonic antigen mRNA in circulating peripheral blood of pancreatic and gastric carcinoma patients. *Life Sci* **59**: 2187–2199

Funaki NO, Tanaka J, Kasamatsu T, Seto S, Kaido T and Imamura M (1997*a*) Hematogenous spreading of hepatocellular carcinoma cells: possible participation in recurrence in the liver. *Hepatology* **25**: 564–568

Funaki NO, Tanaka J, Itami A, Kasamatsu T, Ohshio G, Onodera H, Monden K, Okino T and Imamura M (1997*b*) Detection of colorectal carcinoma cells in circulating peripheral blood by reverse transcription polymerase chain reaction targeting cytokeratin-20 mRNA. *Life Sci* **60**: 643–652

Fuqua SA, Falette NF and McGuire WL (1990) Sensitive detection of estrogen receptor RNA by polymerase chain reaction assay. *J Natl Cancer Inst* **82**: 858–861

Gillespie DH, Cuddy KK, Kolbe T and Marks DI (1994) Dissolve and capture: a strategy for analysing mRNA in blood. *Nature* **367**: 390–391

Mayer B, Johnson JP, Leitl F, Jauch KW, Heiss MM, Schildberg FW, Birchmer W and Funke I (1993*a*) E-cadherin expression in primary and metastatic gastric cancer: down-regulation correlates with cellular dedifferentiation and glandular disintegration. *Cancer Res* **53**: 1690–1695

Mayer B, Jauch KW, Guenthert U, Figdor CG, Schildberg FW, Funke I and Johnson JP (1993*b*) De novo expression of CD44 and survival in gastric cancer. *Lancet* **342**: 1019–1022

Moll R, Franke WW, Schiller DL, Geiger B and Krepler R (1982) The catalog of human cytokeratins: patterns of expression in normal epithelia, tumors and cultured cells. *Cell* **31**: 11–24

Moll R, Loewe, Lauffer J and Franke WW (1992) Cytokeratin 20 in human carcinomas. *Am J Pathol* **140**: 427–447

Moll R, Zimbelmann R, Goldschmidt MD, Keith M, Lauffer J, Kasper M, Koch PJ and Franke WW (1993) The human gene encoding cytokeratin 20 and its expression during fetal development and in gastrointestinal carcinomas. *Differentiation* **53**: 75–93

Pearlman NW, Donohue RE, Stiegman GV, Ahnen DJ, Sedlacek SM and Braun TJ (1987) Pelvic and sacropelvic exenteration for locally advanced or recurrent anorectal cancer. *Arch Surg* **122**: 537–141

Semple TU, Quinn LA, Woods LK and Moore GE (1978) Tumor and lymphoid cell lines from a patient with carcinoma of the colon for a cytotoxicity model. *Cancer Res* **38**: 1345–1355

Taylor I (1996) Liver metastases from colorectal cancer: lessons from past and present clinical studies. *Br J Surg* **83**: 456–460

Togo S, Shimada H, Kubota T, Moossa AR and Hoffmann RM (1995) 'Seed' to 'Soil' is a return trip in metastasis. *Anticancer Res* **15**: 791–794

© Cancer Research Campaign 1998